# Plasmid Identification and Plasmid-Mediated Antimicrobial Gene Detection in Norwegian Isolates

**DOI:** 10.3390/microorganisms9010052

**Published:** 2020-12-27

**Authors:** Abdolrahman Khezri, Ekaterina Avershina, Rafi Ahmad

**Affiliations:** 1 Department of Biotechnology, Inland Norway University of Applied Sciences, 2318 Hamar, Norway; Abdolrahman.khezri@inn.no (A.K.); ekaterina.avershina@inn.no (E.A.); 2 Laboratory or Postgenomic Technologies, Izmerov Research Institute of Occupational Health, 105275 Moscow, Russia; 3 Division of Medical Services-Clinical Microbiology, Inland Hospital, 2609 Lillehammer, Norway; 4 Institute of Clinical Medicine, Faculty of Health Sciences, UiT—The Arctic University of Norway, Hansine Hansens veg 18, 9019 Tromsø, Norway

**Keywords:** *Escherichia coli*, *Klebsiella pneumoniae*, antimicrobial resistance, plasmid, plasmid-mediated genes, horizontal gene transfer

## Abstract

Norway is known for being one of the countries with the lowest levels of antimicrobial resistance (AMR). AMR, through acquired genes located on transposons or conjugative plasmids, is the horizontal transmission of genes required for a given bacteria to withstand antibiotics. In this work, bioinformatic analysis of whole-genome sequences and hybrid assembled data from *Escherichia coli,* and *Klebsiella pneumoniae* isolates from Norwegian patients was performed. For detection of putative plasmids in isolates, the plasmid assembly mode in SPAdes was used, followed by annotation of resulting contigs using PlasmidFinder and two curated plasmid databases (Brooks and PLSDB). Furthermore, ResFinder and Comprehensive Antibiotic Resistance Database (CARD) were used for the identification of antibiotic resistance genes (ARGs). The IncFIB plasmid was detected as the most prevalent plasmid in both *E. coli,* and *K. pneumoniae* isolates. Furthermore, ARGs such as *aph(3″)-Ib*, *aph(6)-Id*, *sul1*, *sul2*, *tet(D)*, and *qnrS1* were identified as the most abundant plasmid-mediated ARGs in Norwegian *E. coli* and *K. pneumoniae* isolates, respectively. Using hybrid assembly, we were able to locate plasmids and predict ARGs more confidently. In conclusion, plasmid identification and ARG detection using whole-genome sequencing data are heavily dependent on the database of choice; therefore, it is best to use several tools and/or hybrid assembly for obtaining reliable identification results.

## 1. Introduction

Antimicrobial resistance (AMR) is the ability of microorganisms to resist antimicrobial treatments, especially antibiotics. Infections due to AMR bacteria are a threat to modern health care and are responsible for an estimated 700,000 and 33,000 deaths/year globally and in Europe, respectively [1]. Recently, the World Health Organization (WHO) published a list of pathogens for which urgent global action is needed [2]. Extended spectrum β-lactamases (ESBL) producing and carbapenem-resistant *Enterobacteriaceae* are among the priority one critical section of the WHO pathogen list. There has been a global rise in infections caused by multi-drug resistant clones of *Enterobacteriaceae*, particularly *Klebsiella pneumoniae* and *Escherichia coli* [3].

AMR can arise through various mechanisms, including mutations of chromosomal genes and the acquisition of antibiotic resistance genes (ARGs) from other strains in a process termed horizontal gene transfer (HGT). It is the sharing of genes through HGT that has largely contributed to the global dissemination of ARGs [4]. The genomes of *E. coli* and *K. pneumoniae* are prone to a mutation in stress, depicting the genetic flexibility to upregulate their natural resistance and acquire foreign determinants through HGT due to mobile genetic elements. These elements, such as plasmids, transposons, integrons, and genomic islands, harbor ARGs [5]. Several plasmids like IncF and IncI1 plasmids are known to carry resistance genes in *E. coli*, *K. pneumoniae,* and other *Enterobacteriaceae* [6]. Additionally, the ColE plasmids, which encode colicins, and have killing activity against different bacteria, are also important plasmids [7]. Broad range resistance plasmids are known to be associated with pathogens; for example, a resistance plasmid from *Enterobacteriaceae* can be transferred to a wide variety of Gram-negative organisms.

Whole-genome sequencing (WGS) is an effective method of tracking the onward transmission of bacteria or resistance plasmid transfer between bacteria. It has made it possible to determine and evaluate an organism’s whole DNA sequence at low costs in a short period of time. It allows for the identification of antimicrobial resistance and the early detection of outbreaks or their epidemiological investigation [8]. Moreover, plasmid assembly and characterization following WGS is a difficult task. This happens because the plasmids tend to contain repeat sequences with sizes greater than sequences generated by sequencing platforms such as Illumina technology (San Diego, CA, USA) [9]. Therefore, the need for in silico plasmid detection has emerged due to the difficulty of plasmid DNA purification if they are longer than 50 kbp [10]. In addition, regarding the need for an efficient plasmid identification tool, ARG databases with comprehensive and accurate gene records are needed to assess AMR prevalence. Although several ARG databases exist, Comprehensive Antibiotic Resistance Database (CARD), and ResFinder are the most effective and have sustainable curation strategies [11]. Recent studies have shown that the hybrid assemblies, which are a combination of Illumina and long-read sequencing (e.g., Oxford Nanopore Technology’s MinION) data, are better at identifying plasmids and ARGs [12]. However, this requires advanced bioinformatic and machine learning methods for WGS data analysis [13,14,15].

Globally, AMR is unevenly distributed. Recently, Klein et al. investigated the drug resistance index (DRI) for 41 countries [16]. They have compared the reported data on antibiotics’ use and their resistance to the treatment of infections caused by microorganisms from the WHO priority list [2]. Norway is among the countries with the lowest DRIs (third lowest), and has a DRI value around four-fold lower than that of the country with the highest DRI; India. However, there is an increasing trend in AMR cases in Norway. For example, the percentage of *E. coli* with ESBL, causing septicemia, has increased ten-fold in the last ten years [17]. There has also been a slightly increased prevalence of ESBLs for *E. coli* (6.6% in 2017 and 6.5% in 2018) and *Klebsiella spp.* (5.3% in 2017 and 6.6% in 2018) [17].

This research has utilized different tools and databases to identify plasmids and predict plasmid-mediated ARGs in both *E. coli* and *K. pneumoniae* isolates. Our results indicate that plasmid identification and ARGs prediction are database/tool dependent. In this regard, a hybrid assembly can be considered an efficient way to identify plasmids and predict plasmid-mediated ARGs.

## 2. Materials and Methods

### 2.1. Sample Collection and Characterization

In this study, *E. coli* and *K. pneumoniae* isolates were collected from blood and urine specimens of Norwegian patients, in collaboration with Oslo University Hospital. The sample overview is in Table 1.

### 2.2. Library Preparation and Whole-Genome Sequencing

The WGS data used in this study are from our recent work, which was performed at Oslo University Hospital [18]. In brief, DNA was isolated from bacteria colonies using QIAamp DNA minikit (Qiagen, Hilden, Germany) following the manufacturer’s instructions and was quantified using Qubit fluorometer (Life Technologies, Carlsbad, CA, USA). The libraries were constructed using the Nextera XT kit (Illumina Ltd., San Diego, CA, USA) according to the manufacturer’s recommendations. The libraries were sequenced in pair-end mode (2 × 300 bp) using the Illumina MiSeq platform at the Norwegian Sequencing Center (Oslo, Norway). Furthermore, to make a hybrid assembly, we sequenced three more isolates (*E. coli* 39, *K. pneumoniae* 23, and 27) using the nanopore and the Illumina MiSeq sequencing platforms. Details regarding library preparation, sequencing, and hybrid assembly have previously been reported [12]. All bioinformatic analyses for both plasmid and hybrid assemblies were identical and performed as described below.

### 2.3. Bioinformatic Analyses of Bacterial Genomic

#### 2.3.1. Quality Control and Trimming of Illumina Sequences

Initially, Illumina and Nanopore reads were quality checked using FastQC (v 0.11.8 for Linux) [19]. Then Illumina adapters were removed, and low-quality reads (Phred below 25) were filtered out using Trimmomatic with default parameters [20]. Before downstream analyses, trimmed reads were again quality checked using FastQC software.

#### 2.3.2. Plasmid Assembly and in Silico Plasmid Identification

Putative plasmid sequences were assembled using plasmid flag in SPAdes (v 3.14.1 for Linux) [21]. General statistics of the assembled putative plasmids was assessed using QUAST (v 4.6.0 for Linux) [22]. Putative plasmid sequences were further confirmed using PlasmidFinder (software version: 2.0.1, database version: 2020-07-13) with minimum identity and coverage of 95% and 60%, respectively [23]. In addition to PlasmidFinder, the identification of putative plasmids was performed using two other methods. First, plasmid reference sequences were downloaded from the PLSDB database [24] and a curated database developed by Brooks et al. [25], hereafter referred to as Brooks. Later, assembled putative plasmids were BLAST searched (sequence identity >95% and word size 28) against downloaded reference plasmids databases. Initially, hits (contigs) with coverage between 30 to 100% were extracted and utilized for the next step. Then only hits with qcov ≥90% (*P*_TRUE_) were considered for downstream analysis. The qcov is unique query coverage per subject, calculated after considering any alignment overlaps between different fragments aligned with that specific subject in the database.

#### 2.3.3. Identification of Plasmids Mediated Antimicrobial Resistance Genes (ARGs)

To identify ARGs hosted by plasmids, the assembled putative plasmids for each isolate were submitted to Resfinder 4.0 [26] and resistance gene identifier tool from Comprehensive Antibiotic Resistance Database—CARD [27]. In both Resfinder and CARD, only hits showing ≥95% identity and ≥98% length coverage were considered as ARGs. Later, only hits sharing the same contig with *P*_TRUE_ were regarded as true plasmid-mediated ARGs (ARG_Plasmid_). Hereafter we refer to ARG_Plasmid-PlasmidFinder_ (meaning ARG and *P*_TRUE_ from PlasmidFinder were found on the same contig), ARG_Plasmid-Brooks_ (meaning ARG and *P*_TRUE_ from Brooks were found on the same contig), ARG_Plasmid-PLSDB_ (meaning that ARG and *P*_TRUE_ from PLSDB were found on the same contig).

## 3. Results

In the current study, putative plasmid sequences and ARGs were identified in silico in *E. coli* and *K. pneumoniae* isolates from Norwegian patients. Furthermore, hybrid assemblies from additional three isolates were also analyzed.

### 3.1. General Statistics of Assembled Plasmid Sequences and Hybrid Assembled Sequences

The general statistics for assembled plasmid sequences and hybrid assembled sequences are shown in Table 2. We observed a higher number and bigger contig size for *E. coli* than *K. pneumoniae* isolates. The GC percentage between *E. coli* and K. pneumoniae was similar, and the N50 values (i.e., the minimum contig length required to cover 50% of the assembled genome sequence) were higher in *E. coli*, indicating larger contig size of plasmids which denotes good quality of assembly. Regarding hybrid assembled isolates, generally, bigger contigs and higher N50 values were observed (Table 2). Interestingly GC percentage was higher in both *K. pneumoniae* isolates compared to the *E. coli* isolate.

### 3.2. In Silico Plasmid Validation

Assembled plasmid sequences were further validated with the PlasmidFinder online tool and using BLASTn against PLSDB and Brooks plasmid database. Using the PlasmidFinder tool, we identified plasmid replicons in 39 (67%) and 11 (25%) of *E. coli* and *K. pneumoniae* isolates. This corresponds to two to three and one to two plasmid replicons per isolate caring plasmids in *E. coli* and *K. pneumoniae,* respectively. (Table S1).

**Figure 1:**
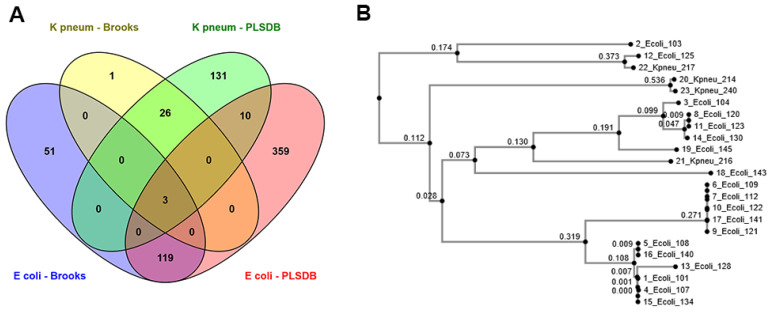
(**A**) Number of uniquely identified putative plasmids (*P*_TRUE_) using different databases after removing duplicates hits per isolate, for both *E. coli* and *K. pneumoniae.* (**B**) phylogenic tree of IncFIB plasmids as the most prevalent plasmids for both *E. coli* and *K. pneumoniae.*

The number of putative plasmids (*P*_TRUE_) after BLASTn and removing duplicates hits per isolate for both *E. coli* and *K. pneumoniae* is shown in Figure 1A. Overall a higher number of *P*_TRUE_ was detected for *E. coli* than *K. pneumoniae* (Table S1). For *E. coli*, the majority of *P*_TRUE_ (122 of 173) detected in the Brooks database were detected using PLSDB as well. In *K. pneumoniae,* almost all the *P*_TRUE_ (29 of 30) detected using the Brooks database were also detected using the PLSDB database. Additionally, we detected 13 shared plasmids between *E. coli* and *K. pneumoniae* using the PLSDB database. In contrast, only three common plasmids were observed between *E. coli* and *K. pneumoniae* by employing the Brooks database. The Neighbor-Joining phylogenetic tree of IncFIB plasmids that were most prevalent for both *E. coli* and *K. pneumoniae* is presented in Figure 1B. The tree is based on MAFFT (Multiple Alignment using Fast Fourier Transform) alignment of plasmids conserved regions [28]. There were four IncFIB sequences of *K. pneumoniae*. Two *K. pneumoniae* isolates formed a separate branch, whereas two others were clustered together with IncFIB plasmid sequences from *E. coli*. An overview of the top 20 most abundant putative plasmids (*P*_TRUE_) and replicons retrieved from each database for both *E. coli* and *K. pneumoniae* is shown in Table 3.

Regarding plasmid detection in hybrid assembled sequences, we managed to retrieve a higher number of *P*_TRUE_ from PLSDB than Brooks and PlasmidFinder. All plasmids detected in Brooks for *E. coli* 39 and *K. pneumoniae* 23 were also detected in the PLSDB database (Table S2). An overview of the top five P_TRUE_ and replicons retrieved from each database for hybrid assembled isolates can be seen in Table 4.

### 3.3. Identification of Plasmid-Mediated ARGs

Plasmid assembled files were used to explore the plasmid-mediated ARGs using ResFinder and CARD databases. As can be seen from Table 5 and using plasmid data, regardless of whether identified ARGs located on the putative plasmids (*P*_TRUE_) or not, we identified more ARGs using the CARD database in *E. coli* isolates compared to ResFinder. For *K. pneumoniae,* opposite results were observed. Moreover, several predicted ARGs were different after annotating the results to plasmid databases (for *E. coli* isolates, ARG_Plasmid-PlasmidFinder_ > ARG_Plasmid-PLSDB_ > ARG_Plasmid-Brooks_ and for *K. pneumoniae* isolates, ARG_Plasmid-PLSDB_ > ARG_Plasmid-PlasmidFinder_ > ARG_Plasmid-Brooks_). Moreover, using hybrid assembled data for *E. coli* 39 isolates, we detected a higher number of ARGs using the CARD database. The same results for *K. pneumoniae* 37 were also observed (Table S2). Surprisingly, no ARGs were detected using *K. pneumoniae* plasmid or hybrid data as ARG_Plasmid-Brooks_.

**Figure 2:**
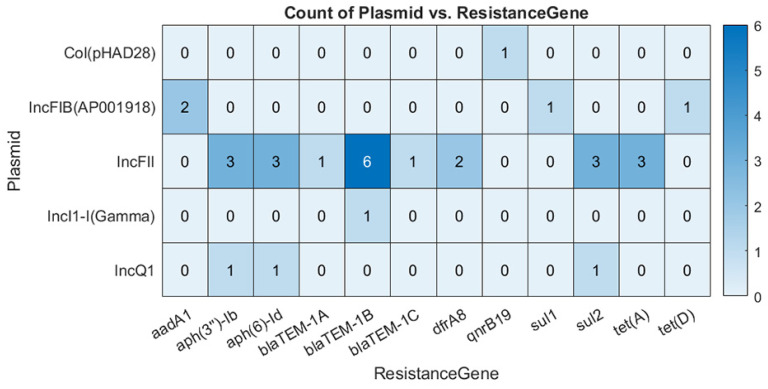
The co-existence of ARG_Plasmid_ genes and different plasmids detected by PlasmidFinder in plasmid data from *E. coli* isolates. Numbers inside each cell indicating the number of isolates where ARGs were found on the corresponding plasmid.

As is apparent from Table 5, ARG_Plasmid-PlasmidFinder_ in *E. coli* plasmid data represent a group with the highest detected number of ARGs. Further details about ARG_Plasmid-PlasmidFinder_ can be seen in Figure 2 and Table 5. For *E. coli* plasmid data, the majority of ARG_Plasmid_ were found on the IncFII plasmid. Furthermore, plasmids such as Col(pHAD28) and IncI1-1(Gamma) hosted the least ARGs. Some ARGs such as *aph(3″)-Ib*, *aph(6)-Id*, *blaTEM-1B,* and *sul2* were carried by more than one type of plasmid.

The most abundant ARG_Plasmid_ genes for plasmid data from *E. coli* and *K. pneumoniae* isolates can be seen in Table 6. The majority of detected ARG_Plasmid_ hits in *E. coli* isolates, carried by *P*_TRUE_ from PlasmidFinder and PLSDB, were beta-lactamase gene-variants blaTEM-1B and TEM-1B. For *E. coli*, ARG_Plasmid_ genes such as *aph(3″)-Ib*, *aph(6)-Id*, *sul1*, *sul2*, and *tet(D)* were flagged as mutual ARG_Plasmid_, observed in all databases. For *K. pneumoniae*, no ARG_Plasmid_ gene was detected on P_TRUE_ from Brooks. However, the *qnrS1* gene was found as a mutual ARG_Plasmid_ harbored by *P*_TRUE_ from both PLSDB and PlasmidFinder.

The ARG_plasmid_ prediction using hybrid assembled sequences is presented in Table 7. Overall, ARG_plasmid_ prediction using hybrid assembled sequences was more consistent between databases compared to plasmid assembled data (Table S2). For instance, in the hybrid assembled *E. coli* 39 isolate, ARG_Plasmid_ such as *aac(3)-VIa* and *aadA1* were hosted by P_TRUE_ from all databases. For *K. pneumoniae* isolate 37, predicted ARG_Plasmid-PlasmidFinder_ and ARG_Plasmid-PLSDB_ were entirely matched. Regarding *K. pneumoniae* isolate 23, besides an extra predicted ARG_Plasmid-PlasmidFinder_, all the predicted ARG_Plasmid-PLSDB_ were covered by ARG_Plasmid-PlasmidFinder_.

## 4. Discussion

In the current research, the applicability of three different plasmid databases and two antibiotics resistance gene databases were assessed using *E. coli* and *K. pneumoniae* assemblies taken from Norwegian patients.

We identified a total number of 490 and 52 exclusive putative plasmids using PLSDB and Brooks databases, respectively. Observed differences might be explained by the content of databases, as the method used for developing the databases and the date of last revision (October 2018 for Brooks and November 2020 for PLSDB) as well as their file size (11,677 and 13,789 entries in Brooks and PLSDB, respectively) are different. Although a BLASTn search against Brooks and PLSDB databases resulted in a higher number of putative plasmids than PlasmidFinder, the method has its disadvantages. For instance, using the BLASTn search, we have detected multiple hits with similar lengths, alignment coverage, and percentage identity for the same assigned contig. Therefore, assigning the putative plasmids as *P*_TRUE_ was challenging. Similar challenges following BLAST+ have been previously described for the FindPlasmid package [29]. Using PlasmidFinder, researchers can directly upload raw files from sequencing platforms. Therefore, de novo assembly is not required, and PlasmidFinder can perform de novo assembly automatically, though the assembly results are not presented by the tool. On the other hand, manual de novo assembly is required in advance to BLASTn search when using other databases such as Brooks and PLSDB. However, one of the disadvantages of using PlasmidFinder is that it currently only covers *Enterobacteriaceae* and a few Gram-positive bacterial species.

It is clinically relevant to perform downstream analyses such as the prediction of plasmid associated ARGs following plasmid identification. In this study, ResFinder performed better than CARD to predict plasmid associated antibacterial resistance genes (AMR_Plasmid-PlasmidFinder,_ AMR_Plasmid-Brooks,_ and AMR_Plasmid-PLSDB_) for both plasmid and hybrid assembled data. In a study comparing the performance of resistance gene databases, both CARD and ResFinder performed equally when submitting a single gene sequence, but CARD performed slightly better for assembled data [30]. Although CARD only accepts FASTA assembly files up to 20 Mb, but in addition to acquired gene information, it contains chromosomal mutation data too. However, ResFinder takes raw files, and assembly is not required. Furthermore, in ResFinder, users can choose between acquired genes or chromosomal mutations. One of the ResFinder advantages is flagging the hit with the true circular term, which indicates whether the hit is plasmid associated or not. Therefore, current data suggest using PlasmidFinder and its associated ResFinder online tools as the first choice to predict plasmid associated ARGs.

In the current study, the AMR_Plasmid_ gene profiles differed between *E. coli* strains carrying plasmids of the same type. Similar results have been reported for *Salmonella entrica* isolates in Ghana [31]. This further highlights the mobility of genetic elements between plasmids, resulting in acquiring or losing the ability for antimicrobial resistance. IncF plasmids are known carriers of a broad spectrum of antibiotic resistance genes in *E. coli* [32,33,34]. In line with this, IncFII plasmids were strongly associated with various resistance genes in our study. These plasmids carried TEM-1B, *aph, sul, tetA,* and *dfr* genes conferring resistance to penicillins, aminoglycosides, sulfonamides, tetracyclines, and trimethoprim [35]. IncFIB were the most prevalent plasmids in our dataset, and they exhibited a low association with antibiotic resistance genes. As such, *aad* (aminoglycoside), *sul* (sulfonamides), and *tet* (tetracycline) were located on IncFIB plasmid contig in two cases, highlighting the low, albeit growing, antibiotic resistance in Norway [16]. However, phylogenetic analysis of IncFIB plasmids revealed that plasmid sequences were shared between *E. coli* and *K. pneumoniae*, probably indicating its ability for inter-species transfer, which raises a concern over rising antibiotic resistance in Norway [17]. Additionally, in this work, we documented the co-existence of blaCTX−M genes with other genes corresponding to resistance against sulfonamide, aminoglycoside, trimethoprim, and tetracycline. This agrees with previous reports indicating that plasmids harboring blaCTX−M genes frequently also carry other genes encoding resistance to other antimicrobials [36,37,38].

The high sequence error rate in Oxford Nanopore Technologies and incongruity between short/fragmented reads from MiSeq Illumina platform and large repetitive regions in plasmids often results in the inaccurate prediction of plasmid-mediated ARGs. To overcome this issue, hybrid assembly has been suggested [12,39]. In the present research, we found that the prediction of ARG_Plasmids_ following hybrid assembly was more consistent across different databases. Having a less fragmented assembly where the circular plasmids are apparent makes the prediction of ARG_plasmid_ more accurate. The current conclusion regarding the applicability of hybrid assembly for plasmid-mediated ARGs detection previously has been made [40]. Therefore, future work implanting hybrid assembly to identify ARGs in bacteria is worth investigating.

## 5. Conclusions

In conclusion, we have demonstrated that plasmid detection and plasmid-mediated ARG prediction are challenging and to obtain a reliable result, one must consider different tools and databases. In the present study, a combination of PlasmidFinder and ResFinder tools showed promising results for both *E. coli* and *K. pneumoniae* isolates. Plasmid detection and prediction of plasmid-mediated ARG can be facilitated using hybrid assembly. Although Norway is considered as a country with a low antibiotic resistance frequency, current research provides a reasonable argument to tackle the slightly increasing antibiotic resistance issue in Norway.

## Figures and Tables

**Table 1:** An overview of the number of clinical isolates collected from Norwegian patients.

Isolates	Source	
	Blood	Urine
***E. coli***	53	5
***K. pneumoniae***	40	4

**Table 2:** An overview of general statistics (mean ± SD) obtained using the QUAST tool for the Scheme 39. and *K. pneumoniae* 23, 37).

	Number of Contigs	Largest Contig (bp)	Total Length (bp)	GC (%)	N50
***E. coli***	87.5 ± 484.8	47,992 ± 65,592	271,880 ± 634,155	48.8 ± 2.3	31,029 ± 62,333
***K. pneumoniae***	7.1 ± 7.9	35,135 ± 48,184	98,095 ± 170,354	48.5 ± 3.8	23,022 ± 29,064
***E. coli* 39**	60	2,757,734	5,955,163	50.51	996,338
***K. pneumoniae* 23**	15	5,305,106	5,831,976	56.75	5,305,106
***K. pneumoniae* 37**	28	2,952,449	5,558,213	57.59	2,952,449

**Table 3:** An overview of the top 20 most abundant putative plasmids (*P*_TRUE_) in both *E. coli* and *K. pneumoniae* retrieved from different databases or tools. (*n*: number of isolates).

	PlasmidFinder		Brooks		PLSDB	
	*Replicon Name*	*n*	*Plasmid Name*	*n*	*Plasmid Name*	*n*
***E. coli***	IncFIB (AP001918)	23	pECO-bc6	5	pMTY17816_OXA48	7
	Col156	15	pUT189	4	pMTY17823_OXA48	7
	IncFIA	9	pUM146	4	p53_E-OXA48	7
	IncFII (29)	9	pRS218	4	pECO-bc6	5
	IncFII	8	pSF-166-1	4	unnamed 7	5
	Col (BS512)	4	pSaT040	4	pUTI89	4
	IncFII (pRSB107)	4	pZH063-1	4	pEC14_114	4
	Col (MG828)	3	pECOS88	3	pUM146	4
	Col8282	2	pMRY16-002_5	3	pRS218	4
	IncFIC (FII)	2	pAPEC-O78-ColV	3	pSF-166-1	4
	IncFII (pCoo)	2	pPSUO78_1	3	unnamed 1	4
	IncI1-I (Gamma)	2	pSF-088-1	3	pSaT040	4
	IncX4	2	pG749_1	3	pZH063_1	4
	Col (pHAD28)	1	pECAZ147_1	3	p0.1229_1	4
	FIA (pBK30683)	1	pECSF1	2	plasmid 2	4
	IncFIB (pB171)	1	pCE10A	2	unnamed	3
	IncM1	1	p3PCN033	2	pF2_18C_Col	3
	IncQ1	1	pMVAST0167_1	2	pDB4277	3
	IncX1	1	pKPN-7c3	2	pCERC4	3
	p0111	1	pEC732_6	2	pCERC5	3
***K. pneumoniae***	IncFIB (K)	4	pKpn23412-4	1	pEC-243	1
	IncFIA (HI1)	2	pK2044	1	pM206-OXA181	1
	IncFIB (pKPHS1)	2	pKCTC2242	1	pM513-OXA181	1
	IncHI1B (pNDM-MAR)	2	PCN061p4	1	pM517-OXA181	1
	repB	2	plasmid B	1	pM518-OXA181	1
	Col (KPHS6)	1	plasmid A	1	pTHC11-1	1
	Col440I	1	pRJF293	1	pTMTA63631	1
	Col440II	1	pRJF999	1	pCA08	1
	ColpVC	1	pNY9_3	1	pKpvK54	1
	IncFIB (AP001918)	1	pESBL931	1	pBH100 alpha	1
	IncFII	1	unnamed 1	1	pVir_095132	1
	IncFII (29)	1	unnamed	1	pSF07201	1
	IncFII (K)	1	unnamed 3	1	pYHCC	1
	IncX3	1	pKp_Goe_579-6	1	pKS22	1
			pKp_Goe_473-5	1	U25P002	1
			pKp_Goe_832-5	1	pFAM22321	1
			pKp_Goe_024-5	1	p183660	1
			pKp_Goe_026-5	1	pKP2442_7c331	1
			pKp_Goe_021-5	1	pOXA-181_29144	1
			pKp_Goe_304-5	1	pEC-NRS18	1

**Table 4:** An overview of the first top-five putative plasmids (P_TRUE_) in hybrid assembled *E. coli* and *K. pneumoniae* isolates retrieved from the different databases.

	Replicon Name from PlasmidFinder	Plasmid Name from Brooks	Plasmid Name from PLSDB
***E. coli* 39**	IncHI2	pCFSAN002069_01	pCFSAN002069_01
	IncHI2A	p25155	p25155
	p0111	PDM02	p2EC1-4
		PDM04	pSH1148_107
			PDM04
***K. pneumoniae* 23**	IncFIB(pNDM-Mar)	pKp_Goe_579-5	p5
	IncFIB(pQil)	pKp_Goe_832-5	pEC25-4
	IncFII(K)	pKp_Goe_304-5	p4_VBA2172
		pKp_Goe_021-5	pRGI01215
		pKp_Goe_026-5	p1_040074
***K. pneumoniae* 37**	IncFII		U25P002
	IncFII(pKP91)		
	IncFIB(K)		
	IncFIA(HI1)		

**Table 5:** Number of total identified ARGs and ARG_Plasmid_ (sharing the same contig with P_TRUE_ from three different databases), using ResFinder and CARD databases in both *E. coli* and *K. pneumoniae* and hybrid assembled isolates. The number inside parentheses shows the percentage of ARG_Plasmid_ identified in P_TRUE_ from different plasmid databases. *E. coli* 39, *K. pneumoniae* 23, and *K. pneumoniae* 37 are hybrid assembled isolates.

	*E. coli*		*K. pneumoniae*		*E. coli* 39		*K. pneumoniae* 23		*K. pneumoniae* 37	
	Res Finder	CARD	Res Finder	CARD	Res Finder	CARD	Res Finder	CARD	Res Finder	CARD
**Total number of ARGs**	61	100	29	15	9	54	24	24	9	17
**PlasmidFinder**	31 (51%)	27 (27%)	1 (3%)	1 (6%)	6 (54%)	6 (11%)	14 (58%)	12 (50%)	6 (54%)	5 (29%)
**Brooks**	10 (16%)	10 (10%)	-	-	2 (22%)	1 (2%)	-	-	-	-
**PLSDB**	18 (29%)	16 (16%)	3 (10%)	2 (13%)	7 (77%)	7 (13%)	7 (29%)	6 (25%)	6 (54%)	5 (29%)

**Table 6:** Gene name and number of isolates with most abundant ARG_Plasmid_ for both *E. coli* and *K. pneumoniae* isolates.

	*E. coli*				*K. pneumoniae*			
	ResFinder		CARD		ResFinder		CARD	
	Gene Name	*n*	Gene Name	*n*	Gene Name	*n*	Gene Name	*n*
**PlasmidFinder**	aadA1	2	aadA	1	qnrS1	1	qnrS1	1
	aph(3″)-Ib	4	aph(3″)-Ib	3				
	aph(6)-Id	4	aph(6)-Id	4				
	blaTEM-1A	1	TEM-1	8				
	blaTEM-1B	7	TEM-40	1				
	blaTEM-1C	1						
	dfrA8	2	dfrA8	2				
	qnrB19	1	qnrB19	1				
	sul1	1	sul1	1				
	sul2	4	sul2	4				
	tet(A)	3	qacEdelta1	1				
	tet(D)	1	tet(D)	1				
**Brooks**	aadA1	1	aadA	1				
	aph(3″)-Ib	2	aph(3″)-Ib	1				
	aph(6)-Id	2	aph(6)-Id	2				
	blaTEM-1B	1	TEM-1	1				
	sul1	1	sul1	1				
	sul2	2	sul2	2				
	tet(D)	1	tet(D)	1				
			qacEdelta1	1				
**PLSDB**	aadA1	1	aadA	1	qnrS1	1	qnrS1	1
	aadA5	1	aadA5	1	floR	1	SHV-1	1
	aph(3″)-Ib	2	aph(3″)-Ib	1	blaSHV-99	1		
	aph(6)-Id	2	aph(6)-Id	2				
	blaTEM-1B	3	TEM-1	3				
	blaTEM-1D	1						
	dfrA17	1						
	mph(A)	1	mph(A)	1				
	sul1	2	sul1	2				
	sul2	2	sul2	2				
	tet(A)	1	qacEdelta1	2				
	tet(D)	1	tet(D)	1				

**Table 7:** Predicted ARG_plasmid_ using hybrid assembled data for both *E. coli* and *K. pneumoniae* isolates.

	*E. coli* 39		*K. pneumoniae* 23		*K. pneumoniae* 37	
	ResFinder	CARD	ResFinder	CARD	ResFinder	CARD
**PlasmidFinder**	aac(3)-VIa		aac(3)-IId	aac(3)-IId	aac(3)-IIa	aac(3)-IIe
	aadA1	aadA	aac(6′)-Ib	aac(6′)-Ib10	aac(6′)-Ib3	
			aac(6′)-Ib-cr		aac(6′)-Ib-cr	
			aadA1	aadA	blaCTX-M-14	CTX-M-14
			aph(3′)-Ia	aph(3′)-Ia	blaTEM-1B	TEM-1
			aph(3″)-Ib	aph(6)-Id	cmlA1	cmlA5
			aph(6)-Id			qacEdelta1
			blaCTX-M-15	CTX-M-15		
			blaOXA-9	OXA-9		
			blaSHV-12	SHV-134		
			blaTEM-1A	TEM-1		
			blaTEM-1B			
			catA1	catI		
			dfrA30	qacE		
			sul2	sul2		
**Brooks**	aac(3)-VIa					
	aadA1					
**PLSDB**	aac(3)-VIa		aac(6′)-Ib	aac(6′)-Ib10	aac(3)-IIa	aac(3)-IIe
	aadA1	aadA	aac(6′)-Ib-cr		aac(6′)-Ib3	
	blaCTX-M-2	CTX-M-2	aadA1	aadA	aac(6′)-Ib-cr	
	blaTEM-1B	TEM-1	blaCTX-M-15	CTX-M-15	blaCTX-M-14	CTX-M-14
	dfrA1	dfrA1	blaOXA-9	OXA-9	blaTEM-1B	TEM-1
	sul1	sul1	blaSHV-12	SHV-134	cmlA1	cmlA5
	tet(A)	qacEdelta1	blaTEM-1A	TEM-1		qacEdelta1

## Data Availability

The data presented in this study are available on request from the corresponding author. The data are not publicly available due to confidentiality agreement related to the AMR-Diag project.

## Supplementary Materials

The following are available online at https://www.mdpi.com/2076-2607/9/1/52/s1, Table S1: an overview of complete results for plasmid assembled data from different databases. Table S2: an overview of complete results for hybrid assembled data from different databases.
